# Assessing the completeness of coded and narrative data from the Victorian Emergency Minimum Dataset using injuries sustained during fitness activities as a case study

**DOI:** 10.1186/s12873-016-0091-4

**Published:** 2016-07-12

**Authors:** Shannon E. Gray, Caroline F. Finch

**Affiliations:** Monash University Accident Research Centre, Monash University, Clayton, Australia; Australian Centre for Research into Sports and its Prevention, Federation University Australia, Ballarat, Australia

**Keywords:** Injury, Surveillance, Quality, Reliability, Emergency department

## Abstract

**Background:**

Injury surveillance systems support the ongoing systematic collection, analysis and interpretation of health information vital to the prevention, planning and evaluation of injury prevention strategies. One key measure of the success of such systems is their reliability. Data completeness is a major component of system reliability, and is an indicator of a system’s data quality. The Victorian Emergency Minimum Dataset (VEMD) is a state-wide record of injury presentations to emergency departments in Victoria, Australia. For each case, it provides information on the injury cause, place of occurrence, activity at time of injury, body region affected and nature of injury, as well as a free-text narrative of the injury event. The aim of this study was to assess the completeness of data in the VEMD using injuries sustained in fitness facilities as a case study.

**Methods:**

Analysis of VEMD coded parent injury variables (nature of injury, injured body region, cause of injury, place where injury occurred, activity at time of injury) and detailed narratives were reviewed for completeness over the ten-year period July 2003 to June 2012, inclusive. Narratives were text analysed manually to determine which items of injury information they contained and compared to the parent injury variables.

**Results:**

There were 2936 identified cases related to injuries sustained during fitness activities. Two percent of cases had all coded injury variables unspecified. Overall, 95.8 % of narratives had at least one piece of injury information missing. The nature of injury and body region variables were coded in 92.6 and 96.6 % of cases, yet were only mentioned in 27.1 and 75.4 % of narratives, respectively. The cause variable was allocated a specified code in 47.7 % of cases and was mentioned in 45.9 % of narratives. The cause was missing in both in 42.8 % of cases. In approximately half of all cases, the activity and place were specified in both the coded injury variable and narrative; they were missing in both in 7.4 and 13.6 % of cases, respectively.

**Conclusions:**

The reliability of the VEMD as an injury surveillance system, varied depending on the injury variable being examined.

## Background

Injury surveillance systems support the ongoing and systematic collection, analysis, interpretation and dissemination of health information [1, 2]. These systems are usually established to provide government and international agencies with data to inform their funding decisions and oversight of health service delivery systems. They are also useful for health care professionals, researchers and the general public because they can provide information on the burden of injuries and the incidence and characteristics of specific injury types [3]. Data collected through such systems can therefore be used to: (i) identify populations at risk of injury; (ii) identify opportunities for intervention, development and implementation; and (iii) evaluate and monitor intervention programs.

In order to reduce the frequency and severity of injuries, the ‘full picture’ of the circumstances of the injury must be known [1]. In particular, it is important to know details about the physical environment where the injury occurred, the activity being participated in at time of injury, whether it was unintentional or intentional in nature, and its aetiology [3, 4]. These factors give important clues as to why injuries occur and, hence, what issues could be addressed to reduce injury risk in the future. Injury surveillance data can be analysed to determine where intervention is necessary, and how injury surveillance systems might be designed, as well as to evaluate the success of prevention programs once implemented [5]. Hence, the severity and types of injuries that occur, and the part of the body most commonly injured, are also useful pieces of information needed to inform the development of injury prevention strategies [4].

In Victoria, Australia, there is currently no universal surveillance system to monitor injuries that occur to people who participate in fitness activities [6, 7]. The only known public source of injuries sustained during fitness activities is the Victorian Emergency Minimum Dataset (VEMD), which is a record of all injury presentations to emergency departments at participating hospitals [6, 7]. Its purpose is to provide necessary epidemiological, health service planning, policy assessment and formulation, clinical research, and quality improvement information to the state government which funds hospital care in Victoria [8]. Data collected in the ED can either be in the form of coded variables, free-text narratives, or a combination of the two [9]. The usefulness of the VEMD in terms of both quality assurance and research relies on its reliability, which can be affected by a number of factors [10]. Injury surveillance conducted in an emergency department is an example of passive surveillance in that relevant information is collected in the course of doing other tasks and not primarily for injury prevention [1].

According to the World Health Organisation (WHO), injury surveillance systems can be assessed and evaluated based on their success across seven attributes: reliability, simplicity, flexibility, acceptability, utility, sustainability and timeliness [1]. The attribute of reliability can be defined as “the ability to collect, manage and provide data properly without failure” [11]. There has been debate regarding whether emergency department injury surveillance data is reliable, as bias can be introduced by factors such as patient age, sex, ethnic origin, time and geographical location [5, 12].

Highly reliable injury surveillance data is needed to ensure accurate estimates of the injury incidence and therefore better estimates of injury risk, as well as providing more detailed information required for development of injury prevention strategies [13]. The development of an epidemiological injury profile and injury prevention strategies could be adversely impacted if relevant cases of a given injury problem are omitted when search criteria or the structure of an injury surveillance system provides incomplete information [14].

The focus of this study was on the completeness (a component of the reliability attribute) of both the coded and narrative (free text) data in the VEMD using data related to injuries sustained during fitness activities as the case study. The completeness of an injury surveillance system reflects its overall quality and can be measured in terms of the proportion of unknown or missing information recorded in key data fields. Higher quality data has a lower level of missing or unknown information surrounding the injury [11]. The specific aim of this study was to evaluate the completeness and quality of a subset of the VEMD as it relates to injuries sustained during fitness activities as a case study. A secondary aim was to assess whether this dataset could be useful for surveillance of injuries sustained during fitness activities, or if its potential use is more aligned only to the needs of emergency department staff who collect the information as part of patient triage.

## Methods

The Victorian Injury Surveillance Unit (VISU) has approval from the Human Research Ethics Committee at the Victorian Department of Health to supply a de-identified subset of data from the Victorian Emergency Minimum Dataset (VEMD). The supplied subset for this study contained cases relating to fitness-related injuries only. The VEMD contains state-wide data on injury presentations to all 39 emergency departments (ED) at public Victorian hospitals that have 24-h access, which has been estimated to record details of approximately 80 % of Victoria’s injury ED presentations [10].

Along with basic demographic and health care information, triage staff at these EDs are required to enter injury characteristic information into six pre-determined coded injury variables for all injury-related presentations (these will herein be referred to as the “parent variables”). They also complete a 250-character-limited narrative that outlines the patient’s personal account of the circumstances leading to their injury in further detail. The six parent variables provide information on the injured *body region*, *nature of injury*, *place* (where injury occurred), *activity* (at time of injury), *human intent* (of the injury) and the *cause* of the injury. For the purposes of this study, the vast majority of injuries sustained at fitness facilities were expected to be unintentional. Therefore, the variable *human intent* was deemed unnecessary and omitted. In order to maintain confidentiality, VISU removed all basic patient demographics and irrelevant information relating to each case prior to providing the subset to authors.

As injuries sustained during fitness activities at fitness facilities were used as the case study, targeted text searching of the narrative was first performed to identify relevant cases. These cases were extracted by VISU and provided to the authors as a data subset as previously reported elsewhere [7]. Examples of fitness-related keywords used to select these cases included: treadmill, elliptical trainer, rowing machine, aerobics, weight training, barbell and dumbbell [6]. This is a select sample given that the narrative had some degree of completeness in order to be selected.

**Fig. 1:**
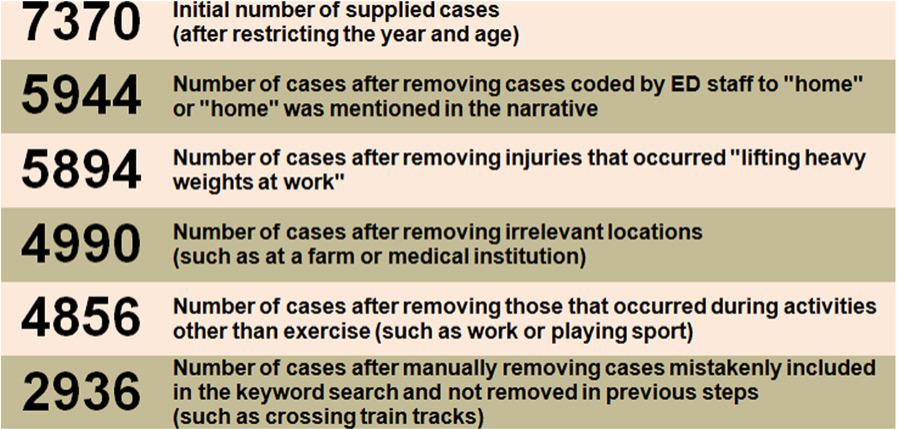
The number of cases following each stage of the associated data cleaning process

Figure 1 shows the steps performed to refine and condense the supplied dataset to remove irrelevant cases that were not related to fitness activities that occurred at fitness facilities, even if they had initially been selected with the text search (e.g. those that occurred at locations or during activities that were clearly not at a fitness facility, such as at home or during work). The initial targeted text search was purposively designed to be very inclusive to ensure high capture of fitness-related cases. The dataset was narrowed to include people aged 15+ years only as most fitness facilities enforce a minimum age limit for membership and use. The dataset was also restricted to the ten-year period July 2003 to June 2012, inclusive.

With the clean dataset, new variables were created alongside the existing (parent) injury variables to categorise the parent injury variables as informative (specified) or uninformative (other specified or unspecified). All cases with missing data were considered ‘unspecified’. Variables originally coded as ‘unspecified’ or ‘other specified’ do not provide any useful information about the injury case and were subsequently deemed uninformative. For example, if the parent *cause* variable was coded as a “fall” the new variable would be coded as informative. It would be coded as uninformative if the parent *cause* variable was coded as ‘unspecified’ or ‘other specified’. It is understood that the timing of the coding of *nature of injury* and *body region* differs between hospitals. These variables can be provisionally coded initially by triage nurses and in certain cases can be updated later, particularly if a procedure is performed on the injured individual as this information is also entered into the VEMD.

According to the VEMD manual, the narrative is intended to identify details not captured by the coded data, and is the patient’s personal account of the injury event. The manual advises including the following information: location, activity, product (specific product involved in the injury, where applicable), and any safety equipment used or absent during the injury occurrence [15]. Nature and cause of injuries are recommended as being additional information that could be included in the narratives but there is no requirement for the injured body region to be mentioned. Nevertheless, each text narrative was coded according to whether it contained each item of information that was also required in the parent injury variables. For example, the new binary variable was coded to 1 (informative) if the text narrative mentioned the affected body region, but coded to 2 (uninformative) if there was no mention of this. As an example, the narrative ‘dislocated shoulder at gym’ provides information about the body region, nature of injury and place at time of injury. However, it does not state what fitness activity was being undertaken at the time of injury (such as lifting dumbbells), nor does it state the cause of the injury (such as the weight was too heavy and the person’s arm gave way).

Data were analysed using SPSS Version 21.0. Descriptive frequency tables of the newly created binary injury variables (informative or uninformative) and narrative specification (narratives state or do not state that particular injury characteristic) were generated for each of the five parent injury variables (*body region*, *nature of injury*, *place*, *activity*, *cause*) to show the proportion of cases that were unspecified. Cross-tabulations were performed for each of the five parent injury variables against the newly created binary injury variables and narrative variables.

Further examination of the text narrative was manually performed to compare the body region mentioned in the narrative directly to what was coded in the parent *body region* variable. If body regions were nearby each other (such as the elbow and upper arm), they were considered to be consistently coded. However, if it was clear that there was a discrepancy (such as a knee was mentioned in the narrative but the injury was coded to the neck in the parent injury variable *body region*), it was considered inconsistent.

## Results

As can be seen in Table 1, fewer than 5 % of cases had a full narrative containing all injury characteristics. In almost two-thirds of cases, at least one injury variable was uninformative (64.6 %). In approximately 2 % of cases, all parent injury variables were uninformative, meaning that to uncover any information about the injury, the narrative needed to be relied upon solely. Of those cases with all parent injury variables unspecified, only one of these had a narrative with complete information. As per the case inclusion criteria, there were no narratives that were completely unspecified, because they would not have been selected in the initial targeted text search.

**Table 1:** The proportion of missing information in narrative and unspecified injury variables reported in the VEMD

	for ANY injury variable		for ALL injury variables	
	n	%	n	%
parent coded data ‘unspecified’	882	30.0	55	1.9
parent coded data uninformative	1896	64.6	56	1.9
narrative missing some detail	2814	95.8	N/A	N/A
narrative missing detail AND parent coded data ‘unspecified’	866	29.5	54	1.8
narrative missing detail AND parent coded data uninformative	1851	63.0	55	1.9

Note: there cannot be all of the narrative unspecified, because the case would not have been selected in the targeted text search inclusion criteria. Column titles apply to the parent coded data. Uninformative includes both ‘unspecified’ and ‘other specified’ parent coded variables

Whilst not necessary for inclusion, the body region injured was mentioned in three-quarters of the narratives (see Table 2). In contrast, the nature of injury was only mentioned in 27.1 % of case narratives. Very few cases were unspecified for the *body region* injury variable (3.4 %), and only 7.4 % of cases were coded as uninformative (‘other specified’ or ‘unspecified’) for the *nature of injury* variable. In contrast, fewer than half of the *cause* variables were coded as informative (47.7 %).

**Table 2:** Completeness of specified parent coding and narratives relating to fitness-related injuries reported in the VEMD

*n* = 2936	Parent coding						Narrative		Informative				Uninformative	
	specified		other specified		unspecified		contained in the text narrative		‘specified’ or ‘other specified’ in parent coding and contained in narrative		‘unspecified’ in parent coding and contained in narrative		‘unspecified’ in parent coding and not contained in narrative	
	n	%	n	%	n	%	n	%	n	%	n	%	n	%
nature of injury	2718	92.6	88	3.0	130	4.4	797	27.1	771	26.3	26	0.9	104	3.5
body region	2835	96.6	N/A^a^	N/A^a^	101	3.4	2214	75.4	2134	72.7	80	2.7	21	0.7
activity	2337	79.6	170	5.8	429	14.6	1796	61.2	1522	51.8	274	9.3	155	5.3
cause	1400	47.7	1039	35.4	497	16.9	1349	45.9	1268	43.2	81	2.7	416	14.2
place	2209	75.2	268	9.1	459	15.6	1898	64.6	1723	58.7	175	6.0	284	9.7

Note: ^a^the *body region* variable does not have ‘other specified’ as an option to select

There were 975 cases (33.2 %) where the body region that was coded in the injury variable did not match to the body region mentioned in the narrative.

**Fig. 2:**
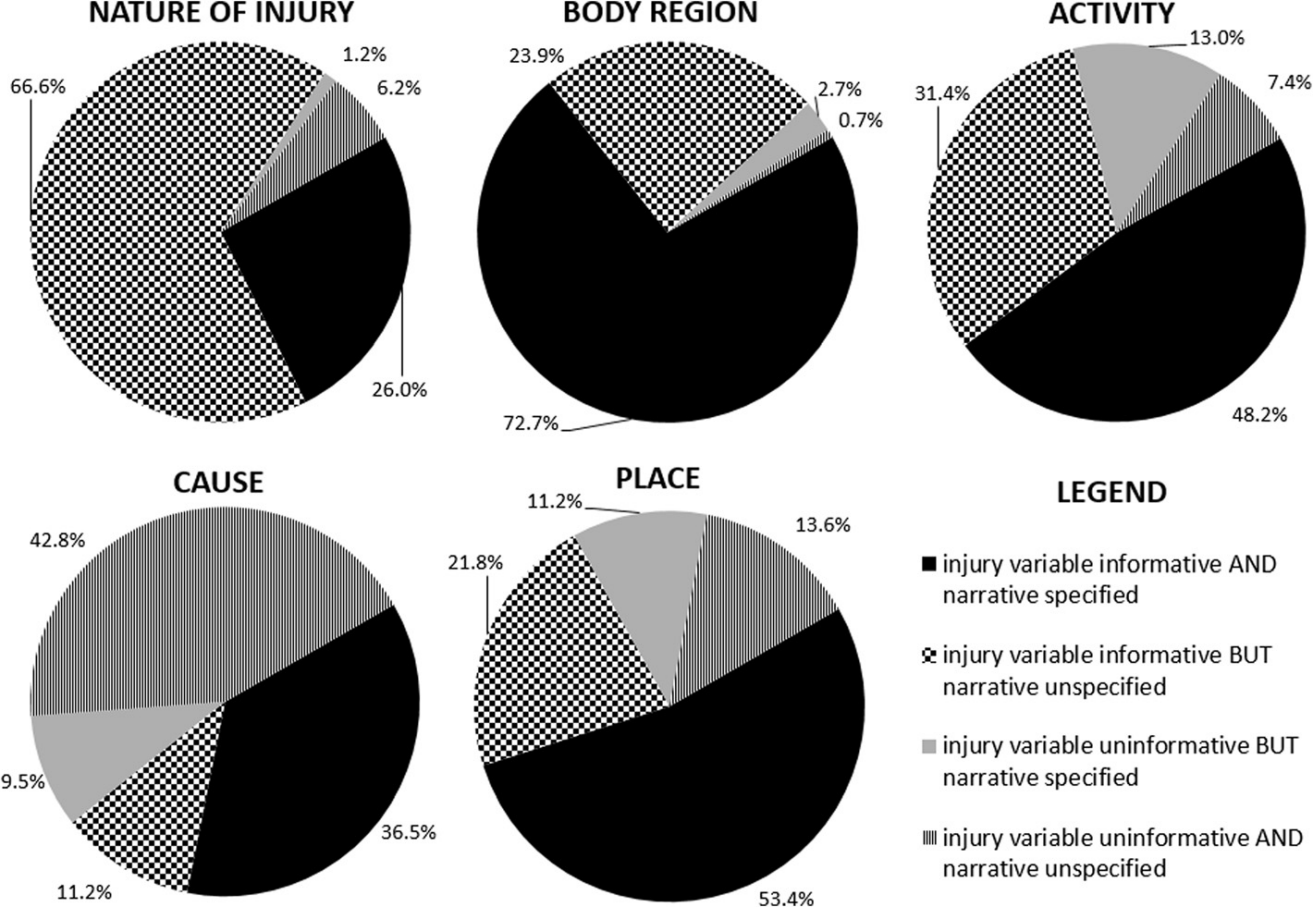
The proportion of specified narratives and informative injury variables for all cases of each of the five injury variables (*n* = 2936 records). Note (for Fig. 2): injury variable informative means that injury variable was ‘specified’ (and uninformative means injury variable was coded as ‘other specified’ or ‘unspecified’). A ‘specified’ narrative contained that injury variable information, conversely an ‘unspecified’ narrative did not contain that information

Figure 2 shows that the parent injury variable and the narrative were jointly specified for more than half of cases for body region and place, and in approximately half of the cases for the activity. For nature of injury, the majority of cases had this detail coded in the parent injury variable, but not mentioned in the narrative. The parent injury variable was not coded nor was the cause of the injury specified in the narrative in 42.8 % of cases, meaning that the causes of injuries associated with fitness activities would be difficult to determine for a large proportion of cases.

## Discussion

Injury surveillance systems are valuable for obtaining, coding and recording specific information surrounding the circumstances of injuries. This study involved an in-depth review of 2936 identified cases of emergency department presentations for treatment of an injury related to fitness activities over a ten-year period July 2003 to June 2012, inclusive. This is only a small proportion of all injury-related ED presentations as there were at least 200,000 per year of the study period [16]. According to the VEMD manual, all injury-related presentations to ED are required to be reported with complete information on all parent injury variables supplemented with a description of the injury event field as a text narrative [8]. When inputting the data into the reporting system, it is necessary for ED triage staff to complete all fields as most systems do not allow incomplete or missing data before a case record is saved. This data can then be altered later if necessary, particularly if further information is added to the case record, such as a procedure. The system does not automatically detect unnecessarily unspecified or uninformative entries and so there is no prompt for further information.

A possible issue with the VEMD, like all ED collected data, is its reliability [10, 17]. Reliability was assessed in this study, as this is the only injury surveillance system attribute that focuses on the data quality; the other six attributes of an injury surveillance system focus on the system itself. In order to evaluate all other attributes, full access to the dataset and data collection methods would be required and should be the focus of other studies.

Our results show that there were some major differences and inconsistencies in what had been written in the narrative, and what was coded in the parent injury variables for body region. Therefore, there are limitations in the completeness of injury characteristics documented in the narrative. There were also a number of cases that either had some of their parent injury variable information missing or were lacking some key information in the narrative. Therefore, each injury case was not always fully recorded and some pertinent information for injury prevention purposes was missing. Unfortunately, this suggests that the VEMD data is not always complete, and there is potential for it to also be inaccurate. Inaccurate data could impact on both the design and development, and the success, of injury prevention strategies if the injury problem is incorrectly represented [10].

For the parent injury variables, the VEMD coding manual specifies which codes to select from, thereby providing a relevant option for all cases without the need to code to unspecified or other specified categories, with the exception of *place* [8]. Referring to the VEMD manual, *body region* and *nature of injury* variable fields provide adequate options to cover all major body parts and types of injuries [8]. As these variables are more likely to be more clinically relevant, this may explain why these were the most successfully coded variables in our case review. There are several broad *cause* of injury categories specified in the VEMD manual that would likely cover the vast majority of injuries sustained during fitness activities, much more than the 47.7 % that were actually specified. The VEMD manual suggests that fitness injuries should be coded to ‘sports’ for *activity*, however triage staff may have assumed that fitness activities do not qualify as a sport, and this could possibly explain why 79.6 % of cases were coded to ‘other specified’ or ‘unspecified’ for *activity*. If this was the case, coding fitness activities to ‘other specified’ is correct, as the activity at the time of injury was specified even if the triage staff member did not deem it appropriate to allocate the case to any pre-defined category. The VEMD manual infers that the place of incidents that lead to injuries at a fitness facility could be coded to either ‘athletics and sports area’ or ‘other specified’ for *place*. This confusion could be responsible for the quarter of cases found to be uninformative for this item, however a higher proportion of uninformative entries were coded to ‘unspecified’ than ‘other specified’. Incompleteness of *activity* and *place* coding for sports injuries has already been shown to lead to underestimates of the true incidence of these injuries in hospital data [14].

While ‘other specified’ is a legitimate possible category for coding variables in datasets with an administrative focus, in terms of injury surveillance it remains uninformative as further information regarding the injury or the injury event cannot be determined using solely coded variables. When parent variables are coded to ‘other specified’, it then becomes necessary to gain further information from the narrative. Unfortunately, such detail is not always provided in the narrative, making it very difficult to determine the full circumstances of the injury. This limits the use of ED data to inform injury prevention efforts fully.

From a treatment point of view, assuming that the information in the injury variables was accurate, injuries still could be treated rather successfully given the information necessary to guide treatment (i.e. injured body region and nature of injury) in the VEMD data was comparatively well coded. Once the data has been collected and recorded, the *activity, place* and *cause* injury variables are unlikely to be referred to again by treating staff within the ED. Knowing this, the triage staff who do the coding may be less inclined to spend much time in accurately completing these data fields, as they neither affect nor aid the patient’s treatment. They may also be unaware of all reasons for why the data is collected and its full range of uses, which could influence their attitude to completing it accurately and completely [10]. It is also possible, that particular software systems within some hospitals may update these fields once a diagnosis has been made and entered into the system (as is required for accurate medical records), without also updating the narrative description of how the condition occurred in the first place.

Around a third of cases mentioned an injured body region in the narrative that did not match with the parent coded body region. The narrative field is the patient’s personal account of the injury events and is recorded by triage staff to clarify the injury event and identify any features not captured by the coded data [8]. This information is necessary for providing additional relevant information related to the injury [8]. Omitting identifying details, the narrative should include the location, activity, specific product being used at time of injury (if appropriate), any safety equipment used, and any additional information such as the nature of the injury and its cause [8]. As the narrative is the patient’s personal account, one would assume that the information provided there is likely to be more accurate than what is coded in the injury variables. However, this may only be in regards to the injury event details, as the triage nurses would potentially have better anatomical and injury knowledge than the patient. Therefore, it is possible that what is written in the narrative is different to what is provided in the actual injury characteristic parent codes if the data field is later updated after treatment in the ED, by medical staff. That being said, injury variable coding is performed by ED triage staff by selecting the most appropriate answer from drop down boxes; data entry mistakes can sometimes be made leading to incorrect data [10]. It would be less likely that triage staff would input text incorrectly than selecting an incorrect option from a drop down box and so it could be expected that the activity at time of injury, place of occurrence and cause of injury would be recorded more successfully in the text narrative. A busy ED or a case requiring urgent medical attention may also affect the level of detail of the triage staff in data input [10, 18].

The VEMD data are collected by a range of ED triage staff (doctors, nurses and clerks) who are not formally trained data coders. It is unknown what level of training is given to these staff members, and whether such education is extensive and includes information on the levels of detail required for the VEMD and what the data are used for, or whether the bare minimum is given [10]. The main responsibility of triage staff is to assess the patient and prioritise their care, and combined with other factors such as their level of training, their attitude to completing injury surveillance tasks and the level of staffing, data quality and its completeness could be lacking [10]. The completeness of the data may have also been affected by other factors such as the number of patients attending the ED, triage status of the patient, time of day, or which hospital was attended. All of these factors could influence the quality of the data collected. A limitation of this study is that, due to ethical considerations the authors were not granted access to the full dataset (for privacy reasons only a subset of the available variables for each case was provided) and therefore these comparisons could not be made.

Being a passive injury surveillance system, the VEMD is simple, practical, affordable, and sustainable [1]. By providing pre-determined drop down boxes for injury variables, and a short text description of the injury event, the dataset easily allows for the collection of useful data during the course of doing other tasks in a busy healthcare environment. Ongoing development of, and improvements to, the VEMD system can be somewhat inflexible as changes require much negotiation between government departments, software developers and administrators of the datasets [10]. It can also be costly to add a new injury variable option as this must be first agreed upon and the entire operating system then updated accordingly within each hospital. Considering the important time-critical work that is performed in EDs, requiring triage staff to spend longer on data entry for each case to ensure more useful data could poorly affect the outcome of patients. It is possible that providing staff with ongoing training and detailed information on how injury data is utilised, could lead to an improvement in the completeness and quality of VEMD data and more efficient data entry by triage staff. Active surveillance, in which injury cases are sought out and investigated, could possibly lead to significantly more reliable and better quality data, but would require significant resources such as funding and staff [1]. It is postulated that more common activities or causes that have pre-determined codes (such as working for income or motor vehicle crashes) could have more reliable data due to the frequency with which they present to EDs compared with fitness activity-related injuries, given they comprise only a small proportion of all ED presentations.

When extracting particular categories of injury cases for detailed review from the VEMD, data extraction is commonly performed using parent injury variables rather than keyword searching of text narratives for particular causes of injury, places where injuries occur or activities at time of injury. Systems where a proportion of these parent variables are coded to unspecified or miscoded values could lead to a vast underestimation of the true magnitude of injury incidence and misrepresentation of the injury problem, which is another consequence of incomplete injury surveillance systems [9].

A limitation of this study was that it only examined whether injury variables were coded as uninformative (‘other specified’ or ‘unspecified’), it did not fully assess the nature of any miscoding. Whilst all cases either occurred at a fitness facility or during an activity most commonly performed at fitness facilities, some injury variables were coded to irrelevant places, activities or causes. Future studies could investigate the degree of miscoding in ED data, assuming the narrative is to be trusted over the coded data [6, 7]. To assess the degree of miscoding in the data fully, each individual case would need to be reviewed with the patient to determine the full circumstances of the injury, and comparisons with recorded data can be made.

The cases represented by the VEMD are likely to vastly underestimate the number of injuries sustained during fitness activities, as a number of injured persons would seek treatment from their general practitioner, allied health professionals, or not at all [6, 7]. Therefore, even if it had 100 % reliability, this dataset would not be appropriate as a sole surveillance system for fitness activity related injuries, as it does not record all injuries sustained. Notwithstanding this, for injuries sustained in fitness facilities, ideally the fitness activity and the cause of the injury need to be provided in the narrative in order for prevention strategies to be developed and implemented.

## Conclusions

The completeness of the VEMD was assessed using injuries that occurred during fitness activities as a case study. Its completeness was found to vary, depending on the injury variable being examined (nature of injury, body region injured, activity when injured, cause of injury, place of occurrence). In more than three-quarters of cases, at least one of the injury variables was uninformative (coded to either ‘unspecified’ or ‘other specified’). The completeness of the narrative varied depending on the injury variable (only around a quarter of cases included the nature of injury, whereas three-quarters of cases included the body region).

According to the WHO injury surveillance guidelines, a reliable system should detect all injury events, fully record these and accurately provide all pertinent information [1]. This study addressed only its completeness. From our results, it is clear that the full circumstances surrounding the injury were not always fully recorded. Moreover, the VEMD gives an underestimation of the injuries sustained in Victoria [10], because not all injury events are able to be identified on the basis of parent coded variables. As this study did not address miscoding, and each injured individual was unable to be contacted to verify the VEMD contents, it is unknown how accurately data were recorded.

Based on the results of this completeness assessment, the VEMD cannot be used as a complete or comprehensive injury surveillance system to monitor fitness activity-related injuries. This study has found that there are gaps in current information systems as not every case provides all injury details in either the coded data or the narrative. In the absence of a universal injury surveillance system to record these, however, the VEMD has the potential to yield useful and important information to still profile the common characteristics of these injuries [6]. Undertaking further detailed analysis of the narratives could potentially yield activity and cause information that is necessary for injury prevention strategies [12, 19], but would be most useful if the details supplied in the text narratives were supplemented with the parent coded data.

## Abbreviations

ED, emergency department; VAED, Victorian admitted episodes dataset; VEMD, Victorian emergency minimum dataset; VISU, Victorian injury surveillance unit
